# More Than Just Movement: Exploring Embodied Group Synchrony During Seated Dance for Older Adults Living in Residential Aged Care Communities

**DOI:** 10.1177/07334648231214946

**Published:** 2023-11-22

**Authors:** Blake Toohey, Marie Hutchinson, Gail Moloney

**Affiliations:** 1 95635 Southern Cross University, Coffs Harbour, NSW, Australia

**Keywords:** dance, music, aged care communities, embodied cognition, synchrony, older adults

## Abstract

One in six people in Australia are aged over 65, with many older adults currently living in residential aged care communities (RACC). Fostering meaningful human connection through social activities, such as dance, is imperative to maintain or improve the quality of life in these settings. Drawing from an embodied cognition framework, this mixed-methods study explored synchrony during a seated dance program with 15 older adults living in a RACC. Qualitative video content analysis was used to code movement, language and music cooccurrences, resulting in five group synchrony labels. Hierarchical Cluster Analysis (HCA) was then employed to distil ten higher-order forms of embodied group synchrony. Using existing neurocognitive evidence, we detail the therapeutic and interpersonal implications of the most prominent forms of embodied group synchrony. These findings can be used to choreograph therapeutic forms of embodied group synchrony in dance programs with older adults.

**What this paper adds**

• A new way to conceptualise group synchrony during dance.
• Confirmation that older adults who experience physical and cognitive impairments are able to engage in a complex array of embodied group synchrony.
• This paper describes the possible functional, cognitive, sensory, social and emotional benefits of embodied group synchrony for older adults in residential aged care facilities.

**Applications of study findings**

• Embodied group synchrony can be easily implemented within existing dance programs allowing dance instructors to potentially work therapeutically with older adults.
• The potential of embodied group synchrony as a non-pharmacological treatment to address specific cognitive and physical impairments.

## Introduction

Increased life expectancy and decreased fertility rates mean that 16% of Australia’s population is now aged over 65 (Australian Institute of Health and Welfare (AIHW), 2023). This increase is reflected in the number of older adults seeking or currently living in residential aged care communities (RACC). RACCs focus on improving residents' quality of life (QOL) by providing medical care, security and social support. However, changes in lifestyle, health and family mean many residents experience feelings of isolation, loneliness and a loss of autonomy (Brownie & Horstmanshof, 2012). Activities that provide meaningful human connections can mitigate these negative feelings.

Dance can provide enjoyable social and physical activity in aged care settings (Britten et al., 2023). Seated dance extends accessibility for those who have varying health needs. Systematic reviews confirm that dance can enhance social interactions (Guzman-Garcia et al., 2012) and foster interpersonal connections between older adults, health workers and family members (Atkins et al., 2019). Meta-analysis reports regular dance participation can improve QOL by providing opportunities to build friendships, reduce depression and maintain social connections (Wu et al., 2021).

Similar non-pharmacological interventions have also demonstrated benefits for older adults. Dance/movement therapy fosters imagination (Capello, 2018), sensory stimulation (Goldstein-Levitas, 2016), embodiment (Capello, 2018), social validation (Shim et al., 2019) and group cohesion (De Tord & Bräuninger, 2015), while decreasing loneliness and negative mood (Ho et al., 2020). Music therapy (Jang-Kunde, 2021) and Tai Chi (Park et al., 2023) have also yielded comparable benefits. A systematic review by Dunphy et al. (2019) found that dance/movement therapy and music therapy effectively decreased depression through various physical, cognitive, emotional and social mechanisms.

The physical benefits of dance are well documented and often linked to the execution of movement. However, is there more to dance than just movement? Dance is a unique activity where movement, music and social interaction simultaneously stimulate the mind and body. Participating in dance requires the complex multisensory and interpersonal integration of proprioceptive, sensorimotor and vestibular control, timed with visual, auditory, social and emotional perceptions, resulting in a unique, multilayered experience of synchrony. Research is yet to fully understand the benefits of synchrony within the experience of dance. This study proposes the question: Can older adults living in RACCs engage in dance that requires complex multisensory synchrony, and if they can, what might the benefits be?

Synchrony emerges when individuals' physiological, emotional or social responses are matched to external or internal cues (Basso et al., 2021). When an individual experiences synchrony, neurobehavioural activity between brain areas synchronises, known as intrabrain synchrony (Basso et al., 2021). The coupling of intrabrain synchrony between two or more individuals during a shared social interaction leads to the individual’s neurobehaviour being ‘in sync’ with each other and is known as interbrain synchrony (Basso et al., 2021). Intrabrain and interbrain synchrony influence physical, emotional, social and affective experiences, illustrating an embodied dynamic between bodily actions, perception and cognition.

Two meta-analyses highlighted that dyadic synchrony enhances positive affect, prosocial behaviour and attitudes (Mogan et al., 2017; Rennung & Göritz, 2016). Prosocial attitudes reflect a desire to help others, while prosocial behaviours involve actions performed for the benefit of others. Rennung and Göritz (2016) found that prosocial behaviour increased when synchrony was intentionally induced, but not prosocial attitudes. Mogan et al. (2017) reported that prosocial behaviour and positive affect positively correlated with group size. Rennung and Göritz (2016) found that group size did not affect prosocial behaviour or attitudes. These conflicting results on the role of group size as a mechanism for a synchrony effect may be due to the different study designs. Rennung and Gortiz (2016) did not provide information on the size of the groups under analysis, while larger studies reviewed by Mogan et al. (2017) focused on neurocognitive rather than affective outcomes.

Apart from Keisari et al. (2022) and Rose et al. (2019), there is limited synchrony research involving older adults. For older adults and people who experience Parkinson’s disease, synchronising movement to music was found to support movement execution (Rose et al., 2019). A recent study reported increased positive affect and perceived social relationships among older adults participating in dyadic synchrony (Keisari et al., 2022), echoing the conclusions drawn from previous meta-analyses with younger populations (Mogan et al., 2017; Rennung & Göritz, 2016). Additional improvements in attention, working memory, concentration and auditory performance were also observed (Keisari et al., 2022).

Currently, no research exists that has explored *group* synchrony for older adults. Older adults typically experience changes in physical and cognitive functioning (De Cock et al., 2018). Therefore, excluding older adults due to physical or cognitive changes limits the generalisability of synchrony research to the older adult population. Further research is needed given the cognitive and affective benefits of dyadic synchrony and the enhanced benefit of prosocial behaviour and positive affect with groups. Investigating the effects of group synchrony for older adults, including those with varying cognitive and physical function, is warranted. Studies demonstrating the benefits of group synchrony have primarily involved younger people and observed motor tasks rhythmically timed to a single cue (Ellamil et al., 2016; Mogan et al., 2017) or been conducted in a laboratory environment. Research in real-world settings is needed.

Synchrony is often conceptualised bimodally, with studies observing the timed execution of movement matched to a single external cue. However, embodied group synchrony encompasses a range of interdependent, simultaneous cognitive, emotional and physiological responses in shared social experiences between two or more people. Group dance offers an opportunity for individuals to engage in embodied group synchrony. However, the different synchronous experiences within group dance have yet to be extensively explored. The synchronisation between different elements of dance, such as movement, music, instructor language, creativity and social interactions, remains understudied. For older adults living in RACCs who experience physical, cognitive and social changes, engaging in embodied group synchrony through dance may provide significant benefits.

While dance has demonstrated positive physical (Liu et al., 2021; Machacova et al., 2017), cognitive (Lossing et al., 2017; Nascimento, 2021), social (Lossing et al., 2017) and QOL (Liu et al., 2021) outcomes for older adults, research has tended to focus on the physical benefits of movement as the catalyst for these other outcomes (Dunphy et al., 2019). Physical synchrony between movement and music results in increased basal ganglia activity (Brown et al., 2006), which is posited as the pathway for observed outcomes (Nascimento, 2021). While the rhythmic cueing of movement to music offers some explanation for the physical and cognitive mechanisms that underlie the benefits of dance, other aspects, such as the interaction with music, language and group members, remain unexplored. The multiple forms of embodied group synchrony that emerge during dance could potentially contribute to these observed benefits.

Using a holistic approach to explore dance, this study addresses the gap in synchrony and dance research among older adults. It aims to explore the nature of embodied group synchrony in a seated dance program with older adults who live in a RACC by identifying the synchronous cooccurrences of movement, language and music. By identifying the combinations and cooccurrences of movement, language and music that generate embodied group synchrony, there is potential to gain a richer understanding of dance and its potential benefits. Moreover, different types of embodied group synchrony may induce distinct physical, emotional, social and sensory experiences among participants, leading to different outcomes.

## Method

### Setting

This study occurred within an Australian RACC during a pre-existing weekly seated dance program delivered by an accredited dance teacher, which was open for all residents to attend. The dance program ran for approximately 50 minutes. The music used varied each week (see supplemental material). The dance program involved participants mirroring the instructor’s movements guided by verbal cues. Approximately 17 residents attended the program.

### Recruitment

All attendees of the dance program were invited to participate in the study. All attendees received verbal and written information about the purpose of the study and the right to participate and withdraw at any time. Eleven females and four males were recruited by providing informed consent in writing to participate. Some participants had a diagnosis of dementia, Alzheimer’s disease, or Parkinson’s disease or used mobility assistance (Table 1). A Human Research Ethics Committee approved this study, approval number 2020/44.

**Table 1.** Participant Demographics.

Demographics			Age		
	*n*	%	*M*	*SD*	Range
Male	4	26.67	85.5	3.87	81–90
Female	11	73.3	89.54	6.9	78–100
Mobile with assistance (rollator walker)	13	86.67	89.53	6.0	81–100
Mobile without assistance	2	13.3	81.5	4.94	78–85
Parkinson’s disease	2	13.3	94	5.65	90–98
Alzheimer’s disease, vascular dementia or other dementia	6	40	86.5	6.44	78–98

### Video-Recorded Participant Observation

Video-recorded participant observation was employed for data collection. This permitted a method of observing the seated dance program and highlighted interactions between participants (Spradley, 2016).

### Data Collection

Two iPads were positioned on either side of the room to capture all participants in the video recordings. Participants were aware of the video recordings. Residents who did not consent to the study were seated out of the frame of the video recordings. The video recordings were uploaded to a computer and combined into one video to enable simultaneous viewing of both frames. Over eight weeks, approximately eight hours of video recordings were collected. Attendance varied over the eight sessions between nine and thirteen participants.

### Data Analysis

Data analysis involved two phases. Phase one used qualitative video content analysis to code movement, instructor language and music in the video footage. Through a reduction process, codes were clustered and labelled as types of group synchrony. Phase two involved subjecting the group synchrony labels to Hierarchical Cluster Analysis (HCA) to derive higher-order embodied group synchrony categories.

#### Qualitative Video Content Analysis Procedure

##### Method

The video was transcribed into narrative text and reduced into meaning units (Schreier, 2012). Each meaning unit comprised three sub-units describing the movement, instructor language and music. Each sub-unit was coded, resulting in a code cluster. Each code cluster was assigned a group synchrony label (Table 2). To mitigate bias, the coding frame was regularly revised by all authors (Schreier, 2012).

**Table 2.** Excerpt of Three Coded Meaning Units.

Time	Meaning Unit			Code Cluster			Group Synchrony Label
	Movement Sub-Unit	Language Sub-Unit	Music Sub-Unit	Movement Codes	Language Codes	Music Codes	
25:38	Slow arm movements come into the chest, then spread wide beside the body	‘And now open up the chest’.	Song has returned to the beginning of a soft, gentle note in the mid-register	Performative Upper Body	Performative Encouraging	Soft Gentle Classical Piano	Performative Group Synchrony
25:47	Rolling arm motion symbolising a rolling brook or other body of running water	‘Rolling brook, the water is rolling’.	Piano music is growing in a gradual crescendo	Performative Symbolic Body	Performative Symbolic	Warm Growing Classical Piano	Performative Group Synchrony
26:05	Arms continuing the rolling motion but rising above the head	No language	Piano music is twinkling and slowing down to an end	Performative Symbolic Body	No Language	Twinkling Classical Piano	Performative Group Synchrony

##### Results

Five group synchrony labels emerged from the qualitative video content analysis and are detailed below.

*1. Performative Group Synchrony.* Performative group synchrony emerged from the timed execution of performative dance movements guided by the instructor’s verbal and physical cues. It was intentionally choreographed but, at times, emerged naturally from the synchronised performance of the group and was creative and expansive in its emergence.
*2. Functional Group Synchrony.* Functional group synchrony stemmed from the execution of practical, functional movements supported by the instructor’s deliberately timed functional verbal guidance.
*3. Affective Group Synchrony.* Affective group synchrony emerged from the movement and language of the instructor that was associated with an emotion or affective experience. These moments of affection were evident in the instructor’s and participants' facial expressions and laughter.
*4. Individual Sensory Synchrony.* Individual sensory synchrony emerged from individual sensory movements, such as hand rubbing and deep breathing performed with music and language that was evocative of sensory perception.
*5. Shared Vocal Synchrony.* Group vocal synchrony emerged when participants' vocalisations were synchronised in timing and pitch under the instructor’s guidance, creating a unified vocal exclamation.

The constitutive codes of each group synchrony label are reported in Table 3.

**Table 3.** Group Synchrony Labels and Their Constitutive Codes.

Group Synchrony Label (*n* = )	Songs	Movement Codes (*n* = )	Language Codes (*n* = )	Music Codes (*n* = )
Performative Group synchrony (139)	• Clair de Lune	• Performative upper body (75)	• Performative encouraging (45)	• Uplifting swing (22)
	• The Infernal Gallop	• Performative symbolic body (39)	• Song lyrics singing (34)	• Uplifting pop (14)
	• Hava Nagila	• Performative complex body (19)	• No language (24)	• Warm growing classical vocals (12)
	• Some Enchanted Evening	• Performative lower body (3)	• Performative symbolic (21)	• Soft gentle pop (11)
	• Every Time You Cry		• Performative metronomic (10)	• Waltzing swing (11)
	• The Rose		• Song harmony singing (2)	• Soft gentle country vocals (9)
	• I Will Survive			• Creeping pop (8)
	• Hernando’s Hideaway			• Harmonising pop vocals (7)
	• Can I Have This Dance			• Waltzing country (7)
	• Dancing with Tears in My Eyes			• Warm growing classical piano (5)
	• Get Me to the Church			• Rising pop vocals (5)
	• When You’re Smilin’			• Twinkling classical piano (4)
				• Soft gentle classical piano (3)
				• Powerful pop vocals (3)
				• Upbeat classical (3)
				• Rising classical vocals (2)
				• Beat-driven classical vocals (2)
				• Rising swing vocals (2)
				• Dramatic classical (2)
				• Suspenseful pop (2)
				• Uplifting country (1)
				• Suspenseful classical (1)
				• Powerful classical vocals (1)
				• Soft gentle classical vocals (1)
				• Tango pop (1)
Functional Group Synchrony (93)	• Clair de Lune	• Functional beat-focused complex body (30)	• Functional metronomic (30)	• Uplifting pop (24)
	• The Infernal Gallop	• Functional beat-focused lower body (20)	• Functional symbolic (17)	• Beat-driven classical vocals (15)
	• Hava Nagila	• Functional beat-focused upper body (16)	• Song lyric singing (11)	• Uplifting swing (15)
	• Some Enchanted Evening	• Functional stretching body (14)	• No language (6)	• Harmonising pop vocals (12)
	• Every Time You Cry	• Functional symbolic body (13)	• Song harmony singing (4)	• Tango pop (5)
	• I Will Survive		• Positive affect statement (3)	• Creeping pop (4)
	• Hernando’s Hideaway			• Uplifting country (3)
	• The Gambler			• Dramatical classical (3)
	• Dancing with Tears in My Eyes			• Warm growing classical vocals (3)
	• Get Me to the Church			• Twinkling classical piano (2)
	• When You’re Smilin’			• Lyrical call and response (2)
				• Soft gentle pop (2)
				• Soft gentle country (2)
				• Soft gentle classical piano (2)
Affective Group Synchrony (29)	• Clair de Lune	• Performative symbolic body (12)	• Positive affect statement (13)	• Uplifting country (10)
	• The Infernal Gallop	• Affective applause (6)	• Symbolic immersive (11)	• Soft gentle country (6)
	• Some Enchanted evening	• Performative upper body (5)	• Symbolic inclusive (2)	• Twinkling classical piano (2)
	• Dancing with Tears in My Eyes	• Affective reciprocal body (2)	• Song lyric singing (3)	• Dramatic classical (2)
	• When You’re Smilin’	• Performative complex body (1)		• Rising swing vocals (2)
	• Get Me to the Church	• Performative humour body (1)		• Uplifting swing (2)
	• The Rose	• Personal sensory body (1)		• Soft gentle pop (1)
	• The Gambler	• Functional stretching body (1)		• Upbeat classical (1)
	• Can I Have This Dance			• Warm growing classical vocals (1)
				• Waltzing country (1)
				• Creeping pop (1)
Individual Sensory Synchrony (11)	• Clair de Lune	• Personal sensory body (11)	• Symbolic sensory (10)	• Soft gentle classical (10)
			• Symbolic immersive (1)	• Twinkling classical piano (1)
Shared Vocal Synchrony (4)	• Clair de Lune	• Performative symbolic body (2)	• Group vocal exclamation (4)	• Dramatic classical piano (2)
		• Performative upper body (2)		• Sudden classical finish (1)
				• Uplifting pop (1)

#### Hierarchal Cluster Analysis Procedure

##### Method

HCA is designed to group data based on similarity (Aldenderfer & Blashfield, 1984) and has been successfully used with data from content analysis (Guest & McLellan, 2003; Henry et al., 2015). In this study, HCA was used to identify groups of cooccurring codes in each group synchrony label and categorise them.

Music codes significantly exceeded the movement and language codes, which would skew the HCA. The music codes were manually integrated into the analysis after the HCA to mitigate this.

The following approach was adopted: (i) identifying categories of movement and language codes within each group synchrony label, (ii) identifying the music codes that cooccurred with the categories, and (iii) integrating the categories and music codes into a final interpretation of embodied group synchrony.

Two group synchrony labels (*Individual Sensory Synchrony* and *Group Vocal Synchrony*) appearance in the data was small enough to interpret without HCA. The remaining three group synchrony labels (*Performative Group Synchrony, Functional Group Synchrony* and *Affective Group Synchrony*) were subjected to HCA.

Each label’s movement and language codes were collated into separate Microsoft Excel spreadsheets. An indicator matrix of the occurrence of codes for each group synchrony label was created. Each indicator matrix was pasted into separate SPSS worksheets. Euclidean Distances were used as the similarity measure, with Ward’s Method as the linkage method. Both techniques have proven effective in other studies with comparable data (Henry et al., 2015). In deciding the final solution, the aim was to derive theoretically meaningful categories, determining the optimal number of categories using dendrograms and agglomeration schedules (Aldenderfer & Blashfield, 1984; Hair & Black, 2000). If the dendrograms and agglomeration schedule did not yield a meaningful solution, category size differences, codes' conceptual distinctiveness and the codes' frequency were examined (Hair & Black, 2000). Any infrequent codes not conceptually relevant were removed, and the HCA was re-run (Hair & Black, 2000).

Following the HCA, the music codes that occurred with each category were identified. Each category’s occurrence was located within the meaning units, noting the song and cooccurring music code. The final step involved integrating the categories and music codes into a final interpretation of synchrony.

##### Results

**Figure 1.**
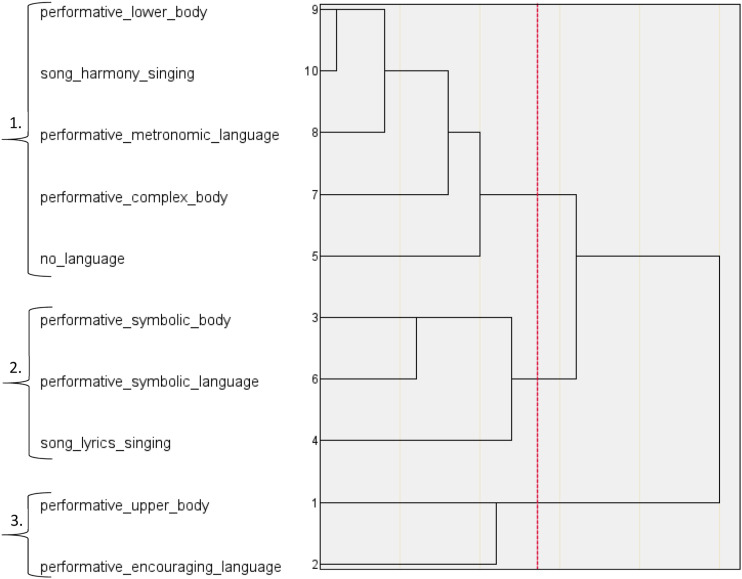
HCA Dendrogram of Performative Group Synchrony; 1. Choreographed Dance Performance Synchrony, 2. Immersive Symbolic Performance Synchrony, 3. Encouraged Upper Body Performance Synchrony.

**Figure 2.**
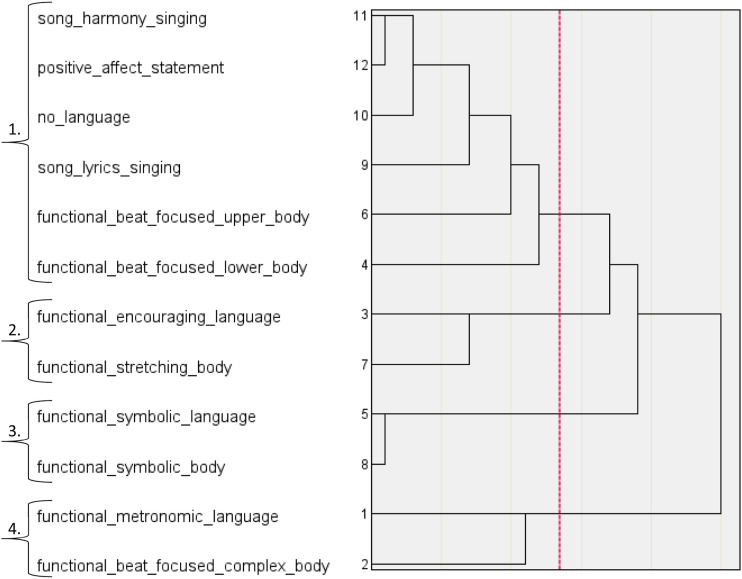
HCA Dendrogram of Functional Group Synchrony; 1. Upper and Lower Body Functional Synchrony, 2. Encouraged Body Stretching Synchrony, 3. Functional Movement Simulation Synchrony, 4. Complex Functional Movement and Language Entrainment Synchrony.

**Figure 3.**
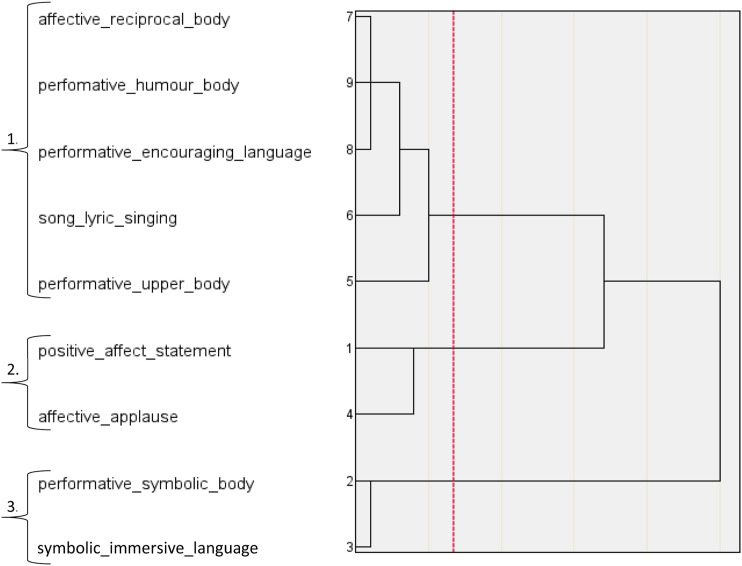
HCA Dendrogram of Affective Group Synchrony; 1. Affective Reciprocal Song and Dance Synchrony, 2. Positive Song Closure Synchrony, 3. Affective Symbolic Performance Synchrony.

The HCAs performed on the three labels of group synchrony distilled ten higher-order categories of embodied group synchrony. For performative group synchrony, the dendrogram (Figure 1) and agglomeration schedule (Table 4) detected three categories reporting a within-cluster homogeneity of 18.838. These categories were labelled *Choreographed Dance Performance Synchrony, Immersive Symbolic Performance Synchrony,* and *Encouraged Upper Body Performance Synchrony.* For functional group synchrony, the dendrogram (Figure 2) and agglomeration schedule (Table 5) detected four categories, reporting a within-cluster homogeneity of 16.830. These categories were labelled *Upper and Lower Body Functional Synchrony, Encouraged Body Stretching Synchrony, Functional Movement Simulation Synchrony, Complex Functional Movement* *and* *Language Entrainment Synchrony.* For affective group synchrony, a subsequent HCA was run after outliers were removed (see supplemental material). The dendrogram (Figure 3) and agglomeration schedule (Table 6) detected three categories, reporting a within-cluster homogeneity of 6.329. The categories were labelled *Affective Reciprocal Song and Dance Synchrony, Positive Song Closure Synchrony* and *Affective Symbolic Performance Synchrony*. See supplemental material for each category’s constitutive movement, language and music codes.

**Table 4.** Agglomeration Schedule for Performative Group Synchrony.

Stage	Cluster Combined		Coefficients	Stage Cluster First Appears		Next Stage
	Cluster 1	Cluster 2		Cluster 1	Cluster 2	
1	9	10	1.118	0	0	2
2	8	9	3.102	0	1	4
3	3	6	5.602	0	0	7
4	7	8	8.385	0	2	5
5	5	7	11.611	0	4	8
6	1	2	15.039	0	0	9
7	3	4	18.838	3	0	8
8	3	5	23.598	7	5	9
9	1	3	30.484	6	8	0

**Table 5.** Agglomeration Schedule for Functional Group Synchrony.

Stage	Cluster Combined		Coefficients	Stage Cluster First Appears		Next Stage
	Cluster 1	Cluster 2		Cluster 1	Cluster 2	
1	11	12	1.323	0	0	3
2	5	8	2.737	0	0	10
3	10	11	4.350	0	1	5
4	3	7	6.472	0	0	9
5	9	10	8.672	0	3	6
6	6	9	11.218	0	5	8
7	1	2	13.957	0	0	11
8	4	6	16.830	0	6	9
9	3	4	20.270	4	8	10
10	3	5	24.002	9	2	11
11	1	3	28.624	7	10	0

**Table 6.** Agglomeration Schedule for Affective Group Synchrony.

Stage	Cluster Combined		Stage Cluster First Appears			Next Stage
	Cluster 1	Cluster 2	Coefficients	Cluster 1	Cluster 2	
1	7	9	.866	0	0	3
2	2	3	1.732	0	0	8
3	7	8	2.687	1	0	4
4	6	7	3.777	0	3	6
5	1	4	5.002	0	0	7
6	5	6	6.329	0	4	7
7	1	5	9.024	5	6	8
8	1	2	12.665	7	2	0

## Discussion

This study explored the nature of group synchrony in a seated dance program for older adults who live in a RACC. Our results demonstrate that seated dance can be choreographed to generate specific types of embodied group synchrony, providing a new way to conceptualise group synchrony.

Theoretically, the synchronicity hypothesis of dance suggests that when individuals synchronise with internal or external stimuli, intrabrain and interbrain synchrony occur within and between individuals (Basso et al., 2021). Intrabrain and interbrain synchrony has been observed through electroencephalogram methods in groups of individuals watching movies (Hasson et al., 2004) and playing music (Sänger et al., 2012). Basso et al. (2021) suggest that intrabrain and interbrain synchrony emerge during group dance from the complex execution of movement in response to shared external and internal sensory perceptions. For older adults, synchronous neurobehaviour associated with dance has important implications.

Our results concur with Basso et al. (2021) and confirm that distinct types of embodied group synchrony can be generated from specific combinations of movement, language and music. Multiple categories of embodied group synchrony were identified, each with unique physical, cognitive, social and emotional characteristics. The variety of embodied group synchrony observed suggests that intrabrain and interbrain synchrony was experienced by the participants. Additionally, our results suggest that the benefits of dance may extend beyond the current acknowledged therapeutic applications of dance that commonly address physical and cognitive functioning for people experiencing Parkinson’s Disease and Alzheimer’s Disease (Lossing et al., 2017; Ruiz-Muelle & López-Rodríguez, 2019). The embodied group synchrony categories with the most important therapeutic implications for older people in RACC are discussed.

### Functional Group Synchrony

Four categories of embodied group synchrony emerged from the functional group synchrony analysis. The category *functional movement simulation synchrony* has the most notable implications for older adults supported by mirror neuron and action observation research. It was characterised by functional movement and functional language with a clear beat.

Mirror neurons are activated in motor domains of the brain during action execution, when watching someone else execute an action (Rizzolatti & Sinigaglia, 2016) or hearing a verbal description of an action (Rizzolatti & Craighero, 2007). Recent neurological research exploring older adults with mild cognitive impairment and Alzheimer’s Disease suggests that the mirror neuron system remains relatively intact, with other areas of the brain compensating for areas in functional decline (Farina et al., 2020). Mirror neurons are the underlying mechanism of action observation therapy, where motor regions of the brain are activated by observing and executing functional actions (Sale et al., 2014). Research has shown that action observation therapy can help rehabilitate motor functions in older adults who have been immobilised after surgery (Marusic et al., 2018), people who have experienced a stroke (Brunner et al., 2014; Franceschini et al., 2010; Sale et al., 2014) and may also help to improve language, motor and social cognition for people with Parkinson’s Disease and Frontotemporal Dementia (Farina et al., 2020; Temporiti et al., 2020). For older adults who engage in choreographed dance, the repeated observation and execution of synchronous functional movement, such as reaching, grasping, pulling and pushing, synchronised with action verbs depicting the performed movement, may contribute to the stimulation and rejuvenation of motor areas in the brain, leading to improvements in motor cognition (Cargnin et al., 2015; Franceschini et al., 2010).

### Individual Sensory Synchrony

*Individual Sensory Synchrony* was characterised by tactile movements such as tapping and rubbing the body accompanied by gentle music, creating sensorimotor and proprioceptive experiences. *Individual sensory synchrony* has possible therapeutic implications for sensory perception and cognition.

It has been suggested that dance can increase brain-derived neurotrophic factor (BDNF) (Kattenstroth et al., 2013). BDNF belongs to a group of proteins known as neurotrophins. Neurotrophins are crucial in brain development, function and plasticity (Notaras & van den Buuse, 2019). BDNF directly affect neurogenesis, synaptic plasticity, and lipid and glucose metabolism within the central and peripheral nervous system (Kattenstroth et al., 2013; Rodziewicz-Flis et al., 2023). Kattenstroth et al. (2013) found that weekly dance programs enhanced the tactile perception of older adults, suggesting these improvements may result from increased BDNF due to the multisensory environment of dance. This is supported by Basso et al. (2021), who suggest that synchronous dance stimulates proprioceptive and sensorimotor systems, enhancing neurobehaviour in sensory brain regions. During the dance program, *individual sensory synchrony* was generated from distinct combinations of external and internal multisensory stimulations of synchronous audio, visual, proprioceptive and somatosensory perceptions. Our results support Kattenstroth et al. (2013) and Basso et al. (2021) and suggest that *individual sensory synchrony* may assist neurobehaviour and BDNF, possibly leading to improved tactile perception for older adults.

### Affective Group Synchrony

Three categories of embodied group synchrony were drawn from affective group synchrony. *Affective symbolic performance synchrony* was the most prevalent, characterised by immersive language and symbolic movement that elicited interactive activities between the participants complemented by music that reflected the created experience, producing a unique emotional and social experience. The synchronous group experiences of positive emotion and social interaction may have significant social and emotional implications for older adults in RACCs.

Dance is an emotional activity that allows self-expression and social interaction through movement, stimulating social and emotional areas of the brain (Basso et al., 2021). The physical interaction between dancers and the perception of music create a rich emotional experience for people participating in and observing dance performances (Vander Elst et al., 2021). Supporting research has demonstrated the joint nature of movement and emotion, where specific movements can influence emotional responses (Koch, 2014), and emotional states can influence movement (Van Dyck et al., 2013). Furthermore, dancing synchronously with a group creates increased positive affect (Mogan et al., 2017).

These social and emotional implications are significant for older adults who live in RACCs where the institutional and clinical approach to care can fail to meet residents' emotional and social needs (Brownie & Horstmanshof, 2012; Meagher et al., 2019). Atkins et al. (2019) suggest dance fosters interpersonal connections for older adults, reporting that community-dwelling older adults who participated in weekly dance programs strengthened social connections with other dance participants, dance instructors, existing friends, healthcare providers and family members (Atkins et al., 2019). Older adults living in RACCs who maintain meaningful relationships are less likely to experience feelings of loneliness and isolation (Brownie & Horstmanshof, 2012), making social and emotional engagement imperative among this population (Gardiner et al., 2020). Dance may be an effective intervention to build social connections between residents, healthcare staff, dance instructors and the family members of participating residents (Atkins et al., 2019). *Affective symbolic performance synchrony* created an environment where participants engaged socially and emotionally with each other, demonstrating the potential power of embodied group synchrony as a therapeutic application and the prospective ability to encourage interpersonal relationships for this population.

### Performative Group Synchrony

Three categories of embodied group synchrony were drawn from performative group synchrony. Additional emotional and social implications are most relevant for the category’s *immersive symbolic performance synchrony* and *choreographed dance performance synchrony*.

*Immersive symbolic performance synchrony* created an experience of interpretive dance. For example, using performative symbolic movements and symbolic language, the instructor created a synchronous group experience of swimming in a lake, gathering sticks and lighting a fire, allowing participants to experience meaningful activities not likely accessible to them presently. Additionally, the category *choreographed dance performance synchrony*, which involved known traditional dance movements like waltzing performed to familiar waltz and country music, allowed participants to experience dancing as a group. Waltz dancing was a popular pastime of this population’s generation and posed an additional invitation to engage in past experiences through physical and auditory reminiscence.

Reminiscence therapy, which involves the retrieval of memories from the past, can be an effective non-pharmacological intervention for addressing depression and improving self-esteem, life satisfaction and emotional well-being for older adults, including those who experience dementia and Alzheimer’s Disease (Cuevas et al., 2020; Melendez Moral et al., 2015). Research has demonstrated that reminiscence therapy that utilises music familiar with past experiences can be a powerful delivery method (Cuevas et al., 2020). Rittiwong et al. (2023) suggest that dance tailored to older adults' cultural backgrounds may promote enjoyment and alleviate depression. From a phenomenological point of view, past experiences are physically stored in the body, and dance has been suggested as a way to elicit memories during reminiscence therapy (Klever, 2013; Skinner, 2010). *Immersive symbolic performance synchrony* and *choreographed dance performance synchrony* used familiar music with symbolic movement and language, inviting the participants to experience activities and events similar to those they may have experienced in the past. Our results suggest that these forms of embodied group synchrony involve reminiscence experiences and may help produce outcomes associated with reminiscence therapy.

### Conclusion

Overall, our results identified the combinations of movement, language and music that act as pathways to five different types of embodied group synchrony and the benefits they may contribute to. This study offers dance instructors a method to tailor their dance programs to achieve specific forms of embodied group synchrony that may support particular cognitive, motor, emotional and social outcomes, providing them a way to work therapeutically with older adults. Our results and supporting research have proposed the distinct types of embodied group synchrony and their constitutive movement, language and music that likely possess the most evident therapeutic applications. Furthermore, when developing dance programs, we encourage instructors to be mindful of diversity within their cohort. Careful selection of music, verbal instructions, movement type and intensity will aid in creating a more inclusive program for people of various cultures, learning styles and abilities.

While dance is often considered an alternative therapy for neurological conditions and as a form of aerobic exercise for older adults, our study suggests broader therapeutic benefits that have largely remained unexplored. This study highlights the significance of recognising dance as an activity with potential therapeutic and interpersonal benefits, especially in addressing critical issues such as loneliness, depression, anxiety and functional changes among older adults in RACCs. The study demonstrates the conceivable potential of embodied group synchrony within dance as a non-pharmacological intervention to enhance brain activity. Acknowledging the therapeutic potential of dance and actively integrating it into the care and treatment plans for older adults in RACCs may contribute to their well-being, social connection and overall QOL. Furthermore, this study advocates for the feasibility of dance to cater to specific symptoms and meet the unique cognitive and physical needs of different groups. This presents an opportunity for gerontologists and healthcare professionals to incorporate dance into their practice and offer it as an alternative to pharmacological treatments. Doing so can provide a more comprehensive and engaging therapeutic experience that encompasses physical and cognitive benefits.

We acknowledge the limitations of this exploratory and novel design, noting that the data yielded was qualitative in origin and subject to confirmation bias. The authors took a reflexive approach to data analysis and caution in result reporting to mitigate this limitation. Nonetheless, this study has highlighted significant gaps and offered exciting new paths to explore within synchrony and dance research.

## Supplemental Material

Supplemental Material - More Than Just Movement: Exploring Embodied Group Synchrony During Seated Dance for Older Adults Living in Residential Aged Care Communities Supplemental Material for More Than Just Movement: Exploring Embodied Group Synchrony During Seated Dance for Older Adults Living in Residential Aged Care Communities by Blake Toohey, Marie Hutchinson and Gail Moloney in Journal of Applied Gerontology.
