# Supplementary material for: More Than Just Movement: Exploring Embodied Group Synchrony During Seated Dance for Older Adults Living in Residential Aged Care Communities
Source: J Appl Gerontol. 2023 Nov 22;43(6):657–69. doi: 10.1177/07334648231214946 (PMC11055412; doi:10.1177/07334648231214946)
Supplement: Supplemental Material - More Than Just Movement: Exploring Embodied Group Synchrony During Seated Dance for Older Adults Living in Residential Aged Care Communities [file sj-pdf-1-jag-10.1177_07334648231214946.pdf]

Categories Drawn from the HCA of Performative Group Synchrony

Performative Group Synchrony				
**Category (*n*=)**	**Movement Codes**	**Language Codes**	**Music Codes**	**Song Occurrence**
Encouraged Upper Body Performance Synchrony (37)	• Performative Upper Body	• Performative Encouraging	• Warm and Growing Classical Piano<br>• Twinkling Classical Piano<br>• Soft Gentle Classical Piano<br>• Upbeat Classical<br>• Rising Vocals<br>• Warm Growing Classical Vocals<br>• Soft Gentle Classical Vocals<br>• Uplifting Pop<br>• Harmonising Pop Vocals<br>• Uplifting Swing<br>• Soft Gentle Pop<br>• Rising Pop Vocals<br>• Powerful Pop Vocals	• Clair De Lune<br>• Every Time You Cry<br>• Hava Nagila<br>• Dancing with Tears in My Eyes<br>• The Rose<br>• The Infernal Gallop<br>• I Will Survive<br>• Some Enchanted Evening
Immersive Symbolic Performance Synchrony (34)	• Performative Symbolic Body	• Performative Symbolic<br>• Song Lyric Singing	• Warm Growing Classical Piano<br>• Twinkling Classical Piano<br>• Upbeat Classical<br>• Suspenseful Classical<br>• Uplifting Swing<br>• Soft Gentle Pop<br>• Powerful Pop Vocals<br>• Uplifting Pop Vocals<br>• Creeping Pop<br>• Suspenseful Pop<br>• Tango Pop	• Clair De Lune<br>• When You're Smilin'<br>• The Rose<br>• The Infernal Gallop<br>• I Will Survive<br>• Hernando's Hideaway
Choreographed Dance Performance Synchrony (9)	• Performative Complex Body<br>• Performative Lower Body	• Performative Metronomic<br>• Song Harmony Singing<br>• No Language	• Beat-Driven Classical Vocals<br>• Waltzing Swing<br>• Uplifting Country<br>• Waltzing Country	• Hava Nagila<br>• Dancing with Tears in My Eyes<br>• When You're Smilin'<br>• Can I Have This Dance

Categories Drawn from the HCA of Functional Group Synchrony

Functional Group Synchrony				
**Category (*n*=)**	**Movement Codes**	**Language Codes**	**Music Codes**	**Song Occurrence**
Complex Functional Movement and Language Entrainment Synchrony (15)	• Functional Beat-Focused Complex Body	• Functional Metronomic	• Harmonising Pop Vocals<br>• Uplifting Pop<br>• Uplifting Swing	• Every Time You Cry<br>• Hava Nagila<br>• Dancing with Tears in My Eyes<br>• When You're Smilin'
Upper and Lower Body Functional Synchrony (10)	• Functional Beat-Focused Upper Body<br>• Functional Beat-Focused Lower Body	• Song Lyric Singing<br>• Song Harmony Singing<br>• Positive Affect Statement<br>• No Language	• Beat-Driven Classical Vocals<br>• Harmonising Pop Vocals<br>• Uplifting Pop<br>• Soft Gentle Pop<br>• Uplifting Country	• Every Time You Cry<br>• Hava Nagila<br>• The Rose<br>• The Gambler<br>• I Will Survive
Functional Movement Simulation Synchrony (11)	• Functional Symbolic Body	• Functional Symbolic	• Beat-Driven Classical Vocals<br>• Uplifting Swing<br>• Uplifting Pop<br>• Creeping Pop<br>• Tango Pop	• Hava Nagila<br>• Get Me to the Church<br>• I Will Survive<br>• Hernando's Hideaway
Encouraged Body Stretching Synchrony (9)	• Functional Stretching Body	• Functional Encouraging	• Twinkling Classical Piano<br>• Warm Growing Classical Vocals<br>• Uplifting Pop<br>• Tango Pop	• Clair De Lune<br>• Every Time You Cry<br>• Some Enchanted Evening<br>• Hernando's Hideaway

Categories Drawn from the HCA of Affective Group Synchrony

Affective Group Synchrony				
**Category (*n*=)**	**Movement Codes**	**Language Codes**	**Music Codes**	**Song Occurrence**
Affective Symbolic Performance Synchrony (10)	• Performative Symbolic Body	• Symbolic Immersive	• Uplifting Country<br>• Soft and Gentle Country<br>• Upbeat Classical	• The Gambler<br>• The Infernal Gallop
Positive Song Closure Synchrony (6)	• Affective Applause	• Positive Affect Statement	• Rising Swing Vocals<br>• Uplifting Swing<br>• Soft and Gentle Pop<br>• Uplifting Country<br>• Warm and Growing Classical Vocals<br>• Waltzing Country	• Dancing with Tears in My Eyes<br>• Get me to the Church<br>• The Rose<br>• The Gambler<br>• Some Enchanted Evening<br>• Can I Have This Dance
Affective Reciprocal Song and Dance Synchrony (5)	• Affective Reciprocal Body<br>• Performative Upper Body<br>• Performative Humour Body	• Performative Encouraging<br>• Song Lyric Singing	• Rising Swing Vocals<br>• Uplifting Swing<br>• Soft Gentle Country<br>• Uplifting Country	• When You're Smilin'<br>• Get Me to The Church<br>• The Gambler

Playlist of Songs

Song	Artist Information	Composed
Clair De Lune	Claude Debussy	1905
Every time You Cry	John Farnham & Human Nature	1997
Hava Naglia	Traditional Jewish Song (composer unknown)	1918
Dancing with Tears in My Eyes	Ruth Etting	1930
Get Me to the Church	Rosemary Clooney (original Frederick Loewe)	1960
When You're Smilin'	Michael Bublé (original Frank Sinatra)	2001
The Rose	Bette Midler	1980
The Gambler	Kenny Rogers	1978
Galop Infernal	Jacques Offenbach	1858
I Will Survive	Gloria Gaynor	1978
Some Enchanted Evening	Enzio Pinza	1949
Hernando's Hideaway	Archie Bleyer	1954
Can I Have This Dance	Anne Murray	1980

Preliminary HCA of Affective Group Synchrony

Employing Euclidean Distances with Wards Method, the initial agglomeration schedule detected three categories reporting a within-cluster homogeneity of 8.773.

Two categories were conceptually relevant. The remaining category contained eight codes and was difficult to interpret conceptually. Upon further examination of the larger eight-code category, the presence of three codes was questioned. *Performative complex body*, *functional stretching body* and *personal sensory body* occurred once within the cluster. *Performative complex body* was better represented in the cluster Performative Group Synchrony, *functional stretching body* was better represented in the cluster Functional Group Synchrony, and *personal sensory body*

was better represented in the group synchrony label *Individual Sensory Synchrony*. It was decided that these codes were not conceptually relevant to this cluster and were excluded from the analysis (Hair & Black, 2000). The HCA was then re-run.

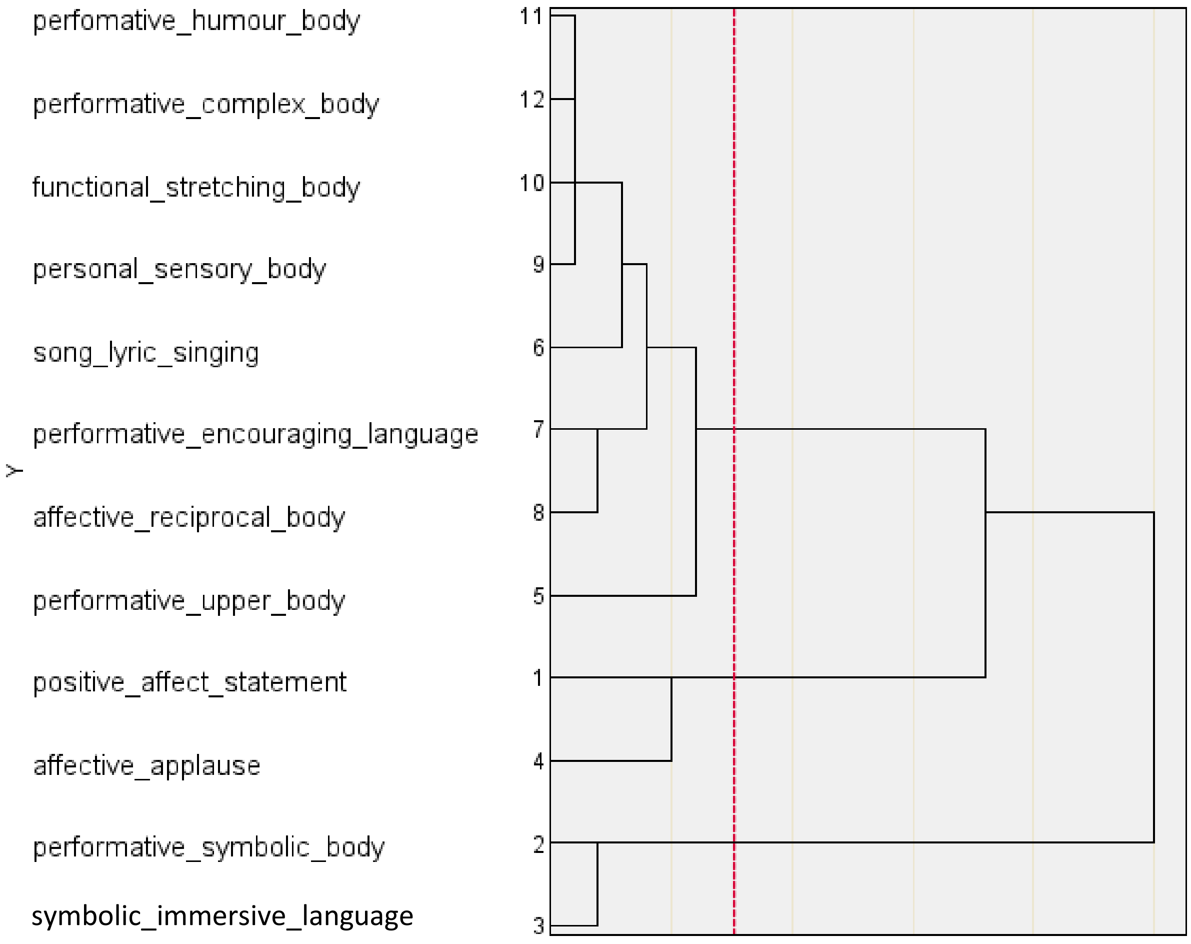

Preliminary HCA of Affective Group Synchrony

Preliminary Agglomeration Schedule for Affective Group Synchrony

Stage	Cluster Combined		Coefficients	Stage Cluster First Appears		Next Stage
	Cluster 1	Cluster 2		Cluster 1	Cluster 2	
1	11	12	.707	0	0	2
2	10	11	1.414	0	1	3
3	9	10	2.121	0	2	6
4	7	8	2.987	0	0	7
5	2	3	3.853	0	0	11
6	6	9	4.912	0	3	7
7	6	7	6.115	6	4	9
8	1	4	7.340	0	0	10
9	5	6	8.795	0	7	10
10	1	5	11.639	8	9	11
11	1	2	15.484	10	5	0
